# Waiting time and mortality rate on lung transplant candidates in Japan: a single-center retrospective cohort study

**DOI:** 10.1186/s12890-021-01760-8

**Published:** 2021-11-29

**Authors:** Takashi Hirama, Miki Akiba, Tatsuaki Watanabe, Yui Watanabe, Hirotsugu Notsuda, Hisashi Oishi, Hiromichi Niikawa, Yoshinori Okada

**Affiliations:** 1 Department of Thoracic Surgery, Institute of Development, Aging and Cancer, Tohoku University Hospital, 4-1 Seiryomachi, Sendai, Miyagi 980-8575, Japan; 2 Division of Organ Transplantation, Tohoku University Hospital, 1-1 Seiryomachi, Sendai, Miyagi 980-8574 Japan

**Keywords:** Lung transplantation, Japan Organ Transplant Network, Waiting time, Mortality, Japan

## Abstract

**Background:**

As lung transplantation (LTX) is a valuable treatment procedure for end-stage pulmonary disease, delayed referral to a transplant center should be avoided. We aimed to conduct a single-center analysis of the survival time after listing for LTX and waitlist mortality in each disease category in a Japanese population.

**Methods:**

We included patients listed for LTX at Tohoku University Hospital from January 2007 to December 2020 who were followed up until March 2021. Pulmonary disease was categorized into the Obstructive, Vascular, Suppurative, Fibrosis, and Allogeneic groups. Risk factors for waitlist mortality were assessed using a Cox proportional hazards model. The Kaplan–Meier method was used to model time to death.

**Results:**

We included 269 LTX candidates. Of those, 100, 72, and 97 patients were transplanted, waiting, and dead, respectively. The median time to LTX and time to death were 796 days (interquartile range [IQR] 579–1056) and 323 days (IQR 129–528), respectively. The Fibrosis group showed the highest mortality (50.9%; *p* < .001), followed by the Allogeneic (35.0%), Suppurative (33.3%), Vascular (32.1%), and Obstructive (13.1%) groups. The Fibrosis group showed a remarkable risk for waitlist mortality (hazard ratio 3.32, 95% CI 2.11–4.85).

**Conclusions:**

In Japan, the waiting time is extremely long and candidates with Fibrosis have high mortality. There is a need to document outcomes based on the underlying disease for listed LTX candidates to help determine the optimal timing for listing patients based on the estimated local waiting time.

**Supplementary Information:**

The online version contains supplementary material available at 10.1186/s12890-021-01760-8.

## Background

Advance in lung transplantation (LTX) has continued to evolve over the last decades and LTX has become a valuable option for selected patients with end-stage pulmonary disease. Delayed referral to a transplant center limits the opportunity for LTX; however, timely referral is difficult for most pulmonary diseases given the limited studies on listed individuals and limited evidence regarding proper consultation for LTX in each pulmonary disease [1, 2]. This issue is more apparent in low-volume transplant centers or countries with severe donor shortages. It is important to describe the populations listed for LTX in each country and establish listing criteria for major pulmonary diseases to accelerate the registration process and improve the equity of transplant opportunities.

Given the limited pool of donated organs, optimizing organ allocation to decrease waitlist mortality in patients listed for LTX is needed. The lung allocation score has decreased the mortality rate among individuals on the waitlist and increased the survival benefit for LTX recipients in the United States [3–5]. However, the allocation system in Japan has remained unchanged in the recent decades. Briefly stated, patients with advanced pulmonary disease are referred to an LTX center for extensive eligibility evaluation, which is reviewed through a two-step assessment at the regional and central committees. Following approval for transplant candidacy, the patients are registered to the Japan Organ Transplant Network (JOTN) and listed at each LTX center. The present system at JOTN allocates cadaveric donor lungs to candidates according to [1] donor age (under age of 18 preferentially to candidates under age of 18), [2] matched lung volume (with the ratio of 0.7–1.3 in the predicted vital capacity), [3] compatible ABO blood type (preferentially identical), and [4] waiting time (preferentially longer candidates on the wait list). Based on the current system that urgency is not considered, the waiting time is crucially considered in recipient selection.

Given the unique circumstances surrounding LTX in Japan, we aimed to conduct a single-center analysis of the survival time after listing for LTX and the waitlist mortality in each disease category. Moreover, we aimed to identify risk factors for mortality in LTX candidates on the waitlist.

## Methods

### Patient population and study objectives

We included patients listed for LTX at Tohoku University Hospital (TUH) from January 1st, 2007 to December 31st, 2020, who were followed up to March 31st, 2021. Data were collected from application forms filled at the time of listing at TUH. Patients who refused LTX after listing or developed non-pulmonary irreversible complications were delisted from the registration file in JOTN. Outcomes (transplanted, dead, and waiting) were reviewed on March 31st, 2021. Pulmonary disease was categorized into obstructive lung disease (Obstructive), pulmonary vascular disease (Vascular), suppurative lung disease (Suppurative), interstitial lung disease (Fibrosis), and allogeneic disease (Allogeneic). The Additional file 1 presents a detailed breakdown of the diseases. The primary objective was to analyze waitlist mortality based on the disease category and to identify risk factors for death. The secondary objective was to evaluate the risk factors for death on the waitlist in patients with idiopathic pulmonary fibrosis (IPF), which is the most common LTX indication in Japan.

### Statistical analysis

Variables were presented as percentages or medians (interquartile range [IQR]), as appropriate. Among-group differences in categorical and continuous variables were compared using the chi-square test and Kruskal–Wallis test, respectively. Further, analyses based on IPF and non-IPF interstitial lung disease (ILD) were performed using the chi-square or Fisher’s exact test and the Mann Whitney test, respectively. Risk factors for waitlist mortality were assessed using a Cox proportional hazards model. Clinically important variables (age, sex, body mass index[BMI]) and presumed risk factors for ILD (forced vital capacity [FVC], connective tissue disorders [CTD], the need of oxygen therapy, 6-min walk distance [6MWD], right ventricular systolic pressure [RVP] measured through transthoracic echocardiogram) were selected for analysis. The Kaplan–Meier method was used to model time to death (survival) where candidates who were waiting on March 31st, 2021 or transplanted in the study period were considered censored. Among-group differences were calculated using the log-rank test. Correlations between the predicted FVC and survival after listing were analyzed using Pearson’s correlation coefficient (r). Statistical significance was set at *p* < 0.05. Statistical analyses and graph generation were performed using EZR (Saitama Medical Center, Jichi Medical University, Saitama, Japan) [6].

## Results

### Overall characteristics of LTX candidates at Tohoku University Hospital

**Fig. 1:**
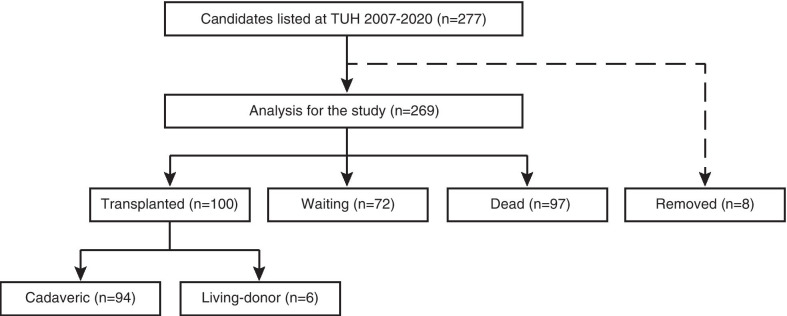
Study flow

Between 2007 and 2020, 1507 patients were registered at JOTN for LTX in the whole of Japan [7], of which 277 patients were applied from TUH, 8 were removed afterward and 269 were analyzed (Fig. 1). Of those, 100 patients were transplanted with either cadaveric- or living-donor while 72 and 97 were waiting and dead, respectively. Table 1 summarizes the characteristics of LTX candidates. The median age was 44 (IQR 31–51), and 50.6% (136/269) of the patients were male. Overall, the predicted FVC was low with a median of 58.0% (IQR 40.9–80.0). The median time to LTX was 796 days (IQR 579–1056) in the 100 transplanted patients; further, time to death was 323 days (IQR 129–528) in 97 patients who passed away on the waitlist.

**Table 1:** Clinical characteristics of the LTX candidates (n = 269) based on the disease category

	Total	Obstructive	Vascular	Suppurative	Fibrosis	Allogeneic	*p *value
	n = 269	n = 61	n = 53	n = 21	n = 114	n = 20	
Age, median (IQR)	44 (31–51)	45 (37–49)	28 (20–38)	47 (39–50)	48 (39–55)	33 (21–47)	< .001
Sex, male (%)	136 (50.6)	17 (27.9)	22 (41.5)	11 (52.4)	77 (53.5)	9 (45.0)	0.017
Body mass index (kg/m2), median (IQR)	19.47 (16.89–23.51)	18.66 (16.68–21.10)	19.57 (17.73–23.50)	18.82 (16.34–20.47)	21.25 (17.19–25.70)	15.90 (14.37–17.32)	< .001
*Blood type, n (%)*							0.1875
A	103 (38.3)	30 (49.2)	18 (33.9)	5 (23.8)	44 (38.6)	6 (30.0)	
O	71 (26.4)	12 (19.7)	11 (20.8)	10 (47.6)	33 (28.9)	5 (25.0)	
B	73 (27.1)	17 (27.9)	17 (32.1)	5 (23.8)	26 (22.8)	8 (40.0)	
AB	22 (8.2)	2 (3.3)	7 (13.2)	1 (4.7)	11 (9.6)	1 (5.0)	
*Pre-transplant condition, n (%)*							
Malignancy	26 (9.7)	2 (3.3)	0 (0.0)	1 (4.8)	10 (8.8)	13 (65.0)	< .001
Connective tissue disorders	47 (17.5)	2 (3.3)	5 (9.4)	2 (9.5)	35 (30.7)	3 (15.0)	0.175
Supplemental oxygen	227 (84.4)	51 (83.6)	46 (86.8)	19 (90.5)	92 (80.7)	19 (95.0)	0.999
*Pulmonary function, median (IQR)*							
Predicted FVC (%)	58.0 (40.9–80.8)	80.7 (58.3–87.7)	83.2 (71.0–94.9)	48.3 (37.6–61.7)	44.5 (30.4–59.7)	43.4 (29.9–50.6)	< .001
6-min walk (m)	300 (199–375)	260 (187–343)	386 (296–376)	261 (187–338)	288 (163–372)	249 (168–345)	< .001
Estimated RVP (mmHg)	38.0 (26.0–59.5)	32.0 (26.0–42.0)	89.5 (73.0–118.0)	38.0 (20.0–61.0)	34.0 (25.0–47.0)	27.5 (22.0–39.5)	< .001
*Laboratory, median (IQR)*							
Hemoglobin (g/dL)	13.6 (12.1–14.9)	13.7 (13.0–14.8)	13.3 (11.8–15.3)	12.1 (11.6–14.6)	13.7 (12.4–15.1)	12.3 (11.1–14.6)	0.127
Albumin (g/dL)	3.9 (3.6–4.2)	4.1 (3.9–4.3)	4.2 (3.8–4.4)	3.5 (3.2–3.8)	3.8 (3.5–4.1)	3.9 (3.6–4.0)	< .001
Creatinine (mg/dL)	0.68 (0.57–0.79)	0.62 (0.53–0.71)	0.70 (0.60–0.80)	0.60 (0.48–0.70)	0.70 (0.59–0.83)	0.55 (0.47–0.80)	0.005
*Outcomes, n (%)*							< .001
Transplanted	100 (37.2)	33 (54.1)	19 (35.8)	11 (52.4)	31 (27.2)	6 (30.0)	
Dead	97 (36.1)	8 (13.1)	17 (32.1)	7 (33.3)	58 (50.9)	7 (35.0)	
Waiting (on March 2021)	72 (26.8)	20 (32.8)	17 (32.1)	3 (14.3)	25 (21.9)	7 (35.0)	
*Wait time, median day (IQR)*							
Time to transplant (n = 100)	796 (579–1056)	981 (678–1132)	959 (602–2015)	758 (676–943)	730 (331–849)	730 (331–849)	0.059
Time to death (n = 97)	323 (129–528)	599 (176–891)	465 (201–897)	196 (5–410)	315 (113–513)	287 (132–449)	0.083
Time to events/censoring (n = 269)	573 (283–991)	930 (538–1193)	959 (483–1804)	685 (203–954)	445 (16–641)	403 (240–640)	< .001

*FVC* forced vital capacity, *IQR* interquartile range, *RVP* right ventricular pressure

### Waitlist mortality in each disease category

The pulmonary disease was categorized as Obstructive, Vascular, Suppurative, Fibrosis, and Allogeneic in 22.7% (61/269), 19.7% (53/269), 7.8% (21/269), 42.4% (114/269), and 7.4% (20/269) of the patients, respectively (Table 1). The following trends were observed: younger age in Vascular (*p* < 0.001), female dominance in Obstructive (*p* = 0.0168), more histories of malignant disease in Allogeneic (*p* < 0.001), higher FVC in Obstructive and Vascular (*p* < 0.001), and lower albumin in Suppurative (*p* < 0.001). Given the disease profiles in each category (Additional file 1), the trends were consistent with the advanced forms of each disease. The Fibrosis group showed the highest mortality at 50.9% (58/114) (*p* < 0.001), followed by the Allogeneic (35.0%), Suppurative (33.3%), Vascular (32.1%), and Obstructive (13.1%) groups. The Suppurative group demonstrated the shortest median time to death (197 days [IQR 5–410]), followed by the Allogeneic (287 days [IQR 132–449]) and Fibrosis (315 days [IQR 113–513]) with no significant difference (*p* = 0.083).

### Analysis of waitlist mortality and its risk factors in LTX candidates at Tohoku University Hospital

**Fig. 2:**
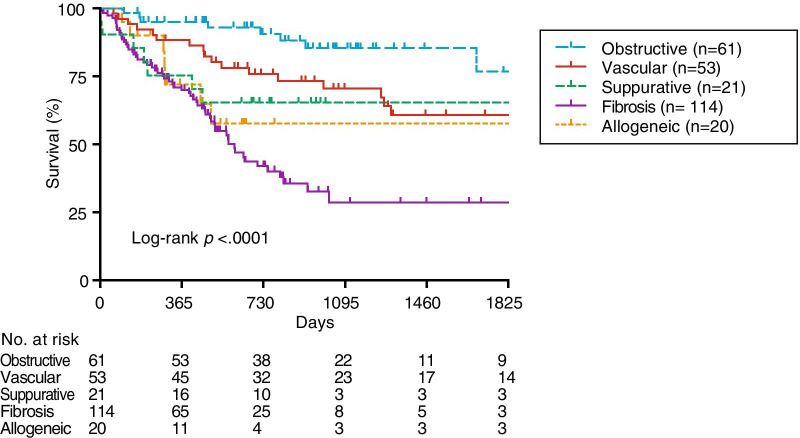
Kaplan–Meier analysis to model time to waitlist mortality in lung transplant candidates among the five disease categories. The number of patients at risk was depicted below the x-axis (days post-listing)

Figure 2 shows the survival in each category. Compared with the other group, Fibrosis had a significantly low survival rate while awaiting LTX (Log-rank *p* < 0.0001). Table 2 presents the risk factors for waitlist mortality in the univariate and multivariate analyses. Fibrosis was a remarkable risk factor for waitlist mortality (HR 3.32, 95% CI 2.11–4.85). Male sex was an independent predictor of death (HR 2.87, 95% CI 1.60–5.16). Age and RVP demonstrated a significant inverse relationship with survival (HR 1.04 per year, 95% CI 1.01–1.07 and HR 1.02 per mmHg, 95% CI 1.01–1.02, respectively). The need of oxygen therapy showed an independent protective effect against waitlist mortality (HR 0.36, 95% CI 0.15–0.86). Patients with lower BMI, smaller FVC, and shorter walk distance did not show increased mortality risks.

**Table 2:** Risk factors for waitlist mortality

	Univariate Cox model			Multivariate Cox model		
	HR	95% CI	*p *value	HR	95% CI	*p *value
Fibrosis (vs. other groups)	3.20	2.11–4.85	0.001	3.39	1.62–7.11	0.001
Age (every 1-year increase)	1.03	1.01–1.05	0.001	1.04	1.01–1.07	0.006
Sex (male vs. female)	2.20	1.45–3.33	< .001	2.87	1.60–5.16	< .001
BMI (every 1 kg/m2 increase)	0.99	0.95–1.03	0.595	0.93	0.86–1.00	0.053
Connective tissue disorders (yes vs. no)	1.00	0.58–1.74	0.992	0.71	0.35–1.47	0.360
Supplemental oxygen (yes vs. no)	0.91	0.52–1.58	0.733	0.36	0.15–0.86	0.021
Predicted FVC (every 1% increase)	0.98	0.97–0.99	< .001	0.99	0.98–1.01	0.428
6-min walk distance (every 1 m increase)	1.00	1.00–1.00	0.032	1.00	1.00–1.00	0.074
Estimated RVP (every 1 mmHg increase)	1.00	0.99–1.01	0.996	1.02	1.01–1.02	0.002

*BMI* body mass index, *CI* confidence interval, *FVC* forced vital capacity, *HR* hazard ratio, *RVP* right ventricular pressure

### Characteristics of LTX candidates with IPF and non-IPF ILD at Tohoku University Hospital

**Fig. 3:**
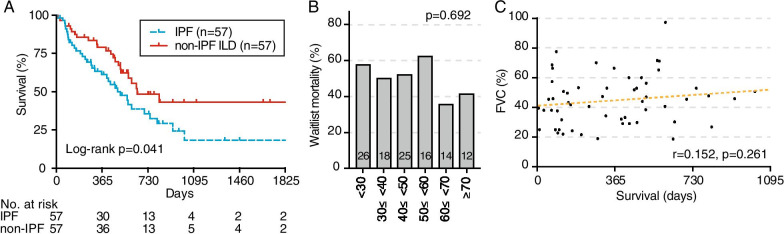
Waitlist mortality of lung transplant candidates with Fibrosis. **a** Kaplan–Meier analysis to model time to waitlist mortality in lung transplant candidates with IPF and non-IPF ILD. The number of patients at risk was depicted below the x-axis (days post-listing). **b** Proportion of waitlist mortality in lung transplant candidates with IPF stratified according to % predicted FVC. The number of candidates in each category is shown in parentheses. **c** Pearson correlation coefficient (r) between the % predicted FVC and survival (time to death).

To determine the specific risk related to the waitlist mortality, the Fibrosis category was further divided into IPF (n = 57) and non-IPF ILD (n = 57). Non-IPF ILD accounted for 50.9% (29/57), 10.5% (6/57), and 8.8% (5/57) of cases of CTD-ILD, drug-induced interstitial lung disease, and pleuroparenchymal fibroelastosis, respectively (Additional file 1). Compared with LTX candidates with non-IPF ILD, those with IPF tended to be senior (50 vs. 47, *p* = 0.039), male (78.9% vs. 56.1%, *p* = 0.016), and die (61.4% vs. 40.4% *p* = 0.039) in the study period (Table 3). However, there was no between-group difference in the predicted FVC at the time of listing (49.0% vs. 43.7%, *p* = 0.314) and overall waiting time (378 vs. 495 days, *p* = 0.267). Compared with non-IPF ILD, IPF was significantly associated with lower survival on the waitlist (Log-rank *p* = 0.41) (Fig. 3A).

**Table 3:** Clinical characteristics of candidates with IPF (n = 57) and non-IPF ILD (n = 57)

	IPF (n = 57)	non-IPF ILD (n = 57)	*p* value
Age, median (IQR)	50 (44–56)	47 (37–52)	0.039
Sex, male (%)	45 (78.9)	32 (56.1)	0.016
Body mass index (kg/m2), median (IQR)	22.6 (18.8–26.2)	19.8 (16.4–24.5)	0.053
*Pre-transplant condition, n (%)*			
History of malignancy	3 (5.3)	7 (12.3)	0.321
Connective tissue disorders	2 (3.5)	33 (57.9)	< .001
Supplemental oxygen	44 (77.2)	48 (84.2)	0.476
*Pulmonary function, median (IQR)*			
Predicted FVC	49.0 (32.9–64.9)	43.7 (29.9–56.8)	0.314
6 min walk (m)	273 (138–369)	300 (203–370)	0.449
Estimated RVP (mmHg)	34 (23–47)	35 (25–47)	0.849
*Laboratory values, median (IQR)*			
Hemoglobin (g/dL)	14.0 (12.6–15.1)	13.5 (12.3–15.0)	0.399
Albumin (g/dL)	3.8 (3.5–4.1)	3.9 (3.6–4.1)	0.444
Creatinine (mg/dL)	0.72 (0.61–0.83)	0.90 (0.55–0.81)	0.116
Outcomes, n (%)			0.039
Transplanted	13 (22.8)	18 (31.6)	
Dead	35 (61.4)	23 (40.4)	
Waiting (on March 2021)	9 (15.8)	16 (28.1)	
*Wait time, median day (IQR)*			
Time to transplant (n = 13, 18)	738 (291–913)	651 (464–850)	0.977
Time to death (n = 35, 23)	278 (98–512)	396 (122–514)	0.623
Time to events/censoring (n = 57, 57)	378 (160–572)	495 (226–646)	0.267

*FVC* forced vital capacity, *ILD* Interstitial lung disease, *IPF* idiopathic pulmonary fibrosis, *IQR* interquartile range, *RVP* right ventricular pressure

### Analysis of waitlist mortality and its risk factors in LTX candidates with Fibrosis

Risk factors for waitlist mortality in IPF were analyzed using a Cox model (Table 4), which revealed that IPF (vs. non-IPF ILD) was an independent predictor of death (HR 1.98, 95% CI 1.11–3.55). Age and male sex showed a significant direct relationship with waitlist mortality (HR 1.07 per 1 year, 95% 1.04–1.10 and HR 2.60, 95% CI 1.29–5.21, respectively). BMI and FVC illustrated an independent negative relationship with risk of death on the waitlist (HR 0.91 per kg/m2, 95% CI 0.84–0.98 and HR 0.97 per % increase, 95% CI 0.95–0.99, respectively). Further analyses revealed that the waitlist mortality due to Fibrosis could not be predicted by the FVC at the time of LTX listing (Fig. 3B); moreover, the survival time of patients with Fibrosis who passed away on the waitlist was not associated with the FVC (Fig. 3C).

**Table 4:** Risk factors for waitlist mortality in patients with IPF

	Univariate Cox model			Multivariate Cox model		
	HR	95% CI	*p *value	HR	95% CI	*p *value
IPF (vs. non-IPF ILD)	1.72	1.02–2.92	0.043	1.98	1.11–3.55	0.022
Age (every 1-year increase)	1.04	1.01–1.06	0.008	1.07	1.04–1.10	< .001
Sex (male vs. female)	4.17	2.36–7.37	< .001	2.60	1.29–5.21	0.007
BMI (every 1 kg/m^2^ increase)	1.00	0.95–1.05	0.856	0.91	0.84–0.98	0.016
Predicted FVC (every 1% increase)	0.97	0.96–0.99	< .001	0.97	0.95–0.99	0.009

*BMI* body mass index, *CI* confidence interval, *FVC* forced vital capacity, *HR* hazard ratio, *ILD* Interstitial lung disease, *IPF* idiopathic pulmonary fibrosis

## Discussion

To our knowledge, this is the first study based on a Japanese transplant center to report the survival time after listing for LTX and the waitlist mortality in each disease category. Waiting time for listed LTX candidates is a common worldwide issue, especially in low-volume centers or regions with an imbalance between organ supply and transplant demand. The median waiting time in our center was 573 days, which was considerably longer than that in other countries[8]; moreover, over one-third of the LTX candidates died after listing. Until December 2018, 668 LTX procedures, including 447 cadaveric-donor and 221 living-donor cases, were performed in Japan, with 18.0% (120/668) of these cases being conducted in TUH [7]. This indicates that all Japanese LTX centers have long waiting times probably due to severe donor shortage (0.61 donations per million) [9]. To alleviate the lack of donated lungs, transplant centers in Japan adopted several policies, including rigorous age limits for listing (under age 55 and 60 for a bilateral and single LTX, respectively); performing single rather than bilateral LTX to maximize LTX opportunities through donor sharing; and providing living-donor transplants for LTX candidates too sick to await cadaveric lungs and with two immediate family members eligible for donating, which accounts for 33.1% (221/668) of all LTX procedures in Japan. As increasing the number of donors could address the long waiting time, close collaboration between all transplant centers and the Japanese government is needed. However, more time is required to address this issue.

Generally, ILD (termed as Fibrosis in this paper) has a poor prognosis; moreover, IPF has a worse prognosis even after listing for LTX [10–14]. At our transplant center, 50.9% and 61.4% of the patients with ILD and IPF, respectively, died while on the waiting list. The consensus from the international society of heart and lung transplantation (ISHLT) proposed listing patients with ILD for LTX after confirming a ≥ 10% drop in FVC or > 50 m decline in 6-min walk test over 6 months [15]. These criteria may be suitable for candidate selection in North America but not for regions with a short donor supply. FVC is routinely measured in respiratory clinics and is among the reliable indicators for assessing disease progression in ILD [16]. Therefore, we validated the probability of waitlist mortality and survival time based on FVC at the time of listing (Fig. 3B and 3C); however, it could not predict them. Therefore, there is a need for studies on credible variables or variable combinations for informing the listing of patients with ILD, especially IPF. Regarding other LTX centers in Japan, a study conducted in Kyoto by Ikezoe et al. reported that shorter 6MWD and lower BMI were independent prognostic factors in candidates with ILD (n = 77) [12]. Moreover, a study conducted in Fukuoka by Miyahara et al. reported that a history of pneumothorax and short 6MWD affected waitlist mortality in patients with ILD [13]. In our study, multivariate Cox analysis revealed that greater age, male sex, and higher RVP were significantly associated with waitlist mortality in Fibrosis. Future studies should assess whether these variables can be used to predict survivors with ILD.

**Fig. 4:**
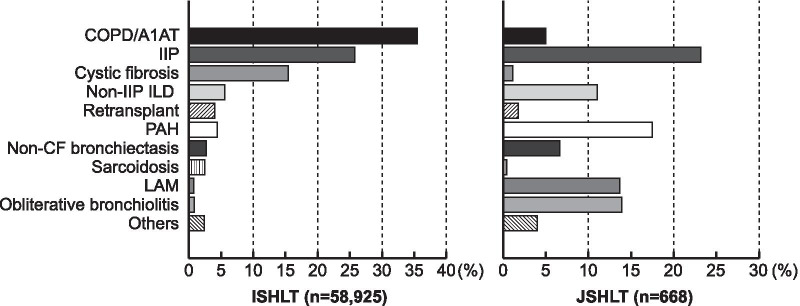
Transplant indications reported from ISHLT and JSHLT. The proportion of transplant indications for adult LTX candidates between January 1995 and June 2017 stratified according to the ISHLT registry report, as well as in both adult and pediatric LTX candidates between January 1998 and December 2018 stratified according to the JSHLT registry report. Reference at ISHLT [24] and JSHLT [7]

To identify risk factors or outcomes in LTX candidates with similar pathophysiology, it would be useful to categorize pulmonary diseases into smaller groups. Pulmonary disease has been classified as A (obstructive disease), B (vascular disease), C (cystic fibrosis), and D (restrictive disease) in the United States, which has improved allocation equity and quality according to the Organ Procurement and Transplantation Network [3, 17]. Since the prevalence of diseases in Japan differs from that in other countries, as well as the little genetic diversity or age limit for listing, we categorized pulmonary diseases based on the transplant circumstance (Additional file 1). Generally, single LTX is primarily considered for the Obstructive and Fibrosis disease categories, as clinically appropriate, while bilateral LTX is inevitably selected for the Vascular and Suppurative disease categories. The Obstructive disease category was mostly comprised of lymphangioleiomyomatosis (LAM). Compared with the ISLHT registry report, there were fewer cases of chronic obstructive pulmonary disease listed in the Obstructive category due to the strict age limit (Fig. 4). Further, bronchiectasis was categorized into the Suppurative, rather than the Obstructive, category since cystic fibrosis is extremely rare in Japan [18, 19]. Compared with other countries, Japan has more cases of pulmonary complications after hematopoietic stem cell transplantation listed for LTX [7, 20]. Since graft-versus-host disease presents with various lung injury phenotypes, including obstructive, restrictive, and mixed [21, 22], it could not simply be categorized into the Obstructive or Fibrosis category. Notably, this phenotypical diversity has been observed in chronic lung allograft dysfunction [23], which could be considered as a host-versus-graft disease from a pathogenesis perspective. To elucidate the risk factors or outcomes in those populations, the Allogeneic category was independently set up and analyzed. There is a need for large-scale studies to determine how to best group LTX indications and to practically assess risk factors for waitlist mortality.

This study has several limitations. First, this was a single-center retrospective study; therefore, our findings do not fully represent the current transplant circumstance in Japan. Some of the patients were listed a decade ago, with the LTX indications having changed over time. Currently, LAM is not often listed while IPF has become increasingly listed and is the most common indication for LTX. Therefore, there is a need for routine reports from the registry organization or academic societies regarding outcomes based on the pulmonary disease listed for LTX. Moreover, there is a need for a prospective study including all transplant centers in Japan to identify risk factors for waitlist mortality in each disease category or specific disease. The timing of referral or listing for LTX should be configured based on the probability of the mortality from the perspective of the waiting time in each region or country. Although our findings are too preliminary to conclude the optimal timing for listing patients with IPF or even the disease category, they could facilitate future multicenter trials.

## Conclusions

Our findings demonstrated that in Japan, the waiting time is exceptionally long and the mortality rate is substantially high among LTX candidates with Fibrosis. There is a need for routine reports documenting the outcomes based on the underlying disease in LTX candidates to help identify prognostic factors in patients on the waitlist, which could allow optimal timing for listing based on the estimated local waiting time. Although our findings are too preliminary to propose a timely listing for each pulmonary disease, our findings could facilitate future related studies.

## Supplementary Information

**Additional file 1**. List of pulmonary disease in transplant candidates in the study.

## Data Availability

The data that support the findings of this study are available on request from the corresponding author. The data are not publicly available due to privacy or ethical restrictions.

## References

[1] Mitchell AB, Glanville AR (2019). Lung transplantation: a review of the optimal strategies for referral and patient selection. Ther Adv Respir Dis.

[2] Shweish O, Dronavalli G (2019). Indications for lung transplant referral and listing. J Thorac Dis.

[3] Valapour M, Lehr CJ, Skeans MA, Smith JM, Miller E, Goff R (2021). OPTN/SRTR 2019 annual data report: lung. Am J Transplant.

[4] Egan TM, Edwards LB (2016). Effect of the lung allocation score on lung transplantation in the United States. J Hear Lung Transplant.

[5] Mooney JJ, Bhattacharya J, Dhillon GS (2019). Effect of broader geographic sharing of donor lungs on lung transplant waitlist outcomes. J Hear Lung Transplant.

[6] Kanda Y (2013). Investigation of the freely available easy-to-use software “EZR” for medical statistics. Bone Marrow Transplant.

[7] The Japanese Society for Heart and Lung Transplantation. http://www2.idac.tohoku.ac.jp/dep/surg/shinpai/.

[8] Stehlik J, Stevenson LW, Edwards LB, Crespo-Leiro MG, Delgado JF, Dorent R (2014). Organ allocation around the world: insights from the ISHLT international registry for heart and lung transplantation. J Hear Lung Transplant.

[9] Transplantation IRIODA. Newsletter June 2021. Barcelona; 2021. https://www.irodat.org/.

[10] Bennett D, Fossi A, Bargagli E, Refini RM, Pieroni M, Luzzi L (2015). Mortality on the waiting list for lung transplantation in patients with idiopathic pulmonary fibrosis: a single-centre experience. Lung.

[11] De Oliveira NC, Julliard W, Osaki S, Maloney JD, Cornwell RD, Sonetti DA (2016). Lung transplantation for high-risk patients with idiopathic pulmonary fibrosis Sarcoidosis. Vasc Diffus lung Dis Off J WASOG.

[12] Ikezoe K, Handa T, Tanizawa K, Chen-Yoshikawa TF, Kubo T, Aoyama A (2017). Prognostic factors and outcomes in Japanese lung transplant candidates with interstitial lung disease. PLoS ONE.

[13] Miyahara S, Waseda R, Tokuishi K, Sato T, Iwasaki A, Shiraishi T (2021). Elucidation of prognostic factors and the effect of anti-fibrotic therapy on waitlist mortality in lung transplant candidates with idiopathic interstitial pneumonias. Respir Investig.

[14] George PM, Patterson CM, Reed AK, Thillai M (2019). Lung transplantation for idiopathic pulmonary fibrosis. Lancet Respir Med.

[15] Weill D, Benden C, Corris PA, Dark JH, Davis RD, Keshavjee S (2015). A consensus document for the selection of lung transplant candidates: 2014—an update from the Pulmonary Transplantation Council of the International Society for Heart and Lung Transplantation. J Hear Lung Transplant.

[16] Du Bois RM, Weycker D, Albera C, Bradford WZ, Costabel U, Kartashov A (2011). Forced vital capacity in patients with idiopathic pulmonary fibrosis: test properties and minimal clinically important difference. Am J Respir Crit Care Med.

[17] Wille KM, Harrington KF, DeAndrade JA, Vishin S, Oster RA, Kaslow RA (2013). Disparities in lung transplantation before and after introduction of the lung allocation score. J Heart Lung Transplant.

[18] Yamashiro Y, Shimizu T, Oguchi S, Shioya T, Nagata S, Ohtsuka Y (1997). The estimated incidence of cystic fibrosis in Japan. J Pediatr Gastroenterol Nutr.

[19] Hirama T, Tomiyama F, Notsuda H, Watanabe T, Watanabe Y, Oishi H (2021). Outcome and prognostic factors after lung transplantation for bronchiectasis other than cystic fibrosis. BMC Pulm Med.

[20] Chambers DC, Cherikh WS, Harhay MO, Hayes D, Hsich E, Khush KK (2019). The international thoracic organ transplant registry of the international society for heart and lung transplantation: thirty-sixth adult lung and heart-lung transplantation report-2019; focus theme: donor and recipient size match. J Heart Lung Transplant.

[21] Hildebrandt GC, Fazekas T, Lawitschka A, Bertz H, Greinix H, Halter J (2011). Diagnosis and treatment of pulmonary chronic GVHD: report from the consensus conference on clinical practice in chronic GVHD. Bone Marrow Transplant.

[22] Greer M, Riise GC, Hansson L, Perch M, Hämmäinen P, Roux A (2016). Dichotomy in pulmonary graft-versus-host disease evident among allogeneic stem-cell transplant recipients undergoing lung transplantation. Eur Respir J.

[23] Glanville AR, Verleden GM, Todd JL, Benden C, Calabrese F, Gottlieb J (2019). Chronic lung allograft dysfunction: definition and update of restrictive allograft syndrome-A consensus report from the Pulmonary Council of the ISHLT. J Hear Lung Transplant.

[24] The International Society for Heart And Lung Transplantation. https://ishlt.org/.

