# Supplementary material for: Waiting time and mortality rate on lung transplant candidates in Japan: a single-center retrospective cohort study
Source: BMC Pulm Med. 2021 Nov 29;21:390. doi: 10.1186/s12890-021-01760-8 (PMC8630869; doi:10.1186/s12890-021-01760-8)
Supplement: Supplementary file 1 — Additional file 1. List of pulmonary disease in transplant candidates in the study. [file 12890_2021_1760_MOESM1_ESM.docx]

**Supplemental data**

Waiting time and mortality rate on lung transplant candidates in Japan: a single-center retrospective cohort study

Takashi Hirama^1,2,¶^, Miki Akiba^2^, Tatsuaki Watanabe^1^, Yui Watanabe^1^, Hirotsugu Notsuda^1^, Hisashi Oishi^1^,

Hiromichi Niikawa^1^, Yoshinori Okada^1,2^

1. Department of Thoracic Surgery, Institute of Development, Aging and Cancer, Tohoku University, Sendai, Miyagi, Japan
2. Division of Organ Transplantation, Tohoku University Hospital, Sendai, Miyagi, Japan

¶ corresponding author

**Supplemental data.** List of pulmonary disease in transplant candidates in the study

| **Obstructive (n=61)**   - Lymphangioleiomyomatosis, 37 - Chronic obstructive pulmonary disease, 17 - Bronchiolitis obliterans, 4 - Other obstructive, 3 |
| --- |

| **Vascular (n=53)**   - Primary pulmonary hypertension/ pulmonary arterial hypertension, 37 - Secondary pulmonary hypertension/ Eisenmenger’s syndrome, 14 - Connective tissue disease-associated-pulmonary hypertension, 2 |
| --- |

| **Suppurative (n=21)**   - Bronchiectasis, 21 (including diffuse panbronchiolitis, 8) |
| --- |

| **Fibrosis (n=114)**   - Idiopathic pulmonary fibrosis, 57 - Connective tissue disease-associated interstitial lung disease, 29 - Drug-induced interstitial lung disease, 6 - Pleuroparenchymal fibroelastosis, 5 - Hypersensitivity pneumonitis, 4 - Nonspecific interstitial pneumonia, 2 - Other fibrosis, 11 |
| --- |

| **Allogeneic (n=20)**   - Graft-versus-host disease post hematopoietic stem cell transplant, 13 - Chronic lung allograft dysfunction, 7 |
| --- |
